# Diagnostic and treatment dilemmas of persistent chronic hypokalaemia in a patient with anorexia nervosa: a case report

**DOI:** 10.1186/1752-1947-8-294

**Published:** 2014-09-05

**Authors:** Kevin Mohee, Katarzyna Kucharska-Pietura, Amalan Karthigeyan, Asif Naqvi

**Affiliations:** 1 Rharian Fields, Specialist Eating Disorder Unit, The Gardens, 2nd Avenue, Grimsby DN33 1NU, UK; 2 Gastroenterology Department, Diana Princess of Wales Hospital, Scartho Road, Grimsby DN33 2BA, UK; 3 Institute of Psychiatry and Neurology, Sobieskiego 9, 02-957 Warsaw, Poland

## Abstract

**Introduction:**

Anorexia nervosa is an eating disorder that continues to have a devastating impact on the lives of both adolescents and adults worldwide. We present a case of persistent chronic hypokalaemia in a patient with anorexia nervosa.

**Case presentation:**

A 26-year-old Caucasian woman was admitted to our eating disorders unit under Section 2 of Mental Health Act (1983) with a body mass index of 13.5kg/m^2^. On admission, biochemical tests highlighted moderate hypokalaemia (3.0mmol/L). She was immediately put on two tablets of Sando-K® (potassium supplement; potassium bicarbonate and potassium chloride twice daily. An electrocardiogram showed signs of bradycardia with T-wave inversion in at least two chest leads. Throughout her admission, she exhibited a fluctuating potassium level (2.5 to 5.0mmol/L). Once on the unit, she was put on Sando-K® (potassium supplement; potassium bicarbonate and potassium chloride) but her potassium still tended to drop. On one occasion when she became normokalaemic, supplementation was stopped and she was put on observations. After 24 hours of 1 to 1 observation by ward staff including fluid input and output monitoring as well as search of her bedroom, she was again found to be hypokalaemic in spite of no evidence to suggest laxative or diuretic abuse.

**Conclusions:**

She kept reiterating to clinicians that her potassium depletion is hereditary, not associated with any purging behaviour and for that reason long-term administration of Sando-K® (potassium supplement; potassium bicarbonate and potassium chloride) tablet remained essential. Self-denial was continued until her planned discharge; however, positive behavioural change was recorded whilst her blood showed normokalaemia for a whole week until her discharge.

## Introduction

Anorexia nervosa (AN) is an eating disorder that continues to have a devastating impact on the lives of both adolescents and adults worldwide. It is a psychiatric disorder with severe metabolic effects on the entire body and according to Thomas Insel, Director of National Institute of Mental Health, USA, AN is the third most common chronic illness among adolescents; AN does not discriminate on the basis of age, gender, race or class and no one is immune [1]. AN has an average prevalence of 0.3 to 1% in women and 0.1% in men for the diagnosis in developed countries [2].

We present a case of persistent chronic hypokalaemia in a patient with AN-binge eating/purging type according to *Diagnostic and Statistical Manual of Mental Disorders*, Fifth Edition (International Statistical Classification of Diseases and Related Health Problems, 10th revision, ICD10-F50.0).

## Case presentation

A 26-year-old Caucasian woman was admitted to our eating disorders unit under Section 2 of Mental Health Act (1983) with a body mass index (BMI) of 13.5kg/m^2^ (weight 30.5kg, height 1.5m). On admission, biochemical tests highlighted moderate hypokalaemia (3.0mmol/L). She was immediately put on two tablets of Sando-K® (potassium supplement; potassium bicarbonate and potassium chloride) twice daily. An electrocardiogram (ECG) showed signs of bradycardia with T-wave inversion in at least two chest leads. She had been experiencing amenorrhea for 9 months prior to admission.

On initial mental state examination, she was cooperative, articulate and maintained good eye contact. She was able to give a coherent chronological account of her story and was fully oriented in time, place and person. She described her mood as fine but objectively appeared low. She denied any suicidal thoughts or plans, deliberate self-harm issues and any psychotic phenomena. Furthermore, she was adamant that she did not present with any abnormal non-delusional thought processes of either anorexic or obsessive-compulsive nature. On admission she had very limited insight into her mental condition and was unable to recognise both physical and psychological effects of malnutrition but accepted the implemented meal plans.

She did not approve of her eating disorder diagnosis but reported her symptoms were driven by weight and shape problems and compliance with meals was not a major issue. Her personal history began at 14-years old when she weighed 89kg and was subsequently bullied. She then started to restrict her diet by consuming alcohol as her main caloric intake and was subsequently vomiting. At the age of 16, her weight dropped to 54kg (BMI 23.9kg/m^2^). She became depressed and stopped attending school. She was admitted to hospital after complaining of abdominal pain. After discharge, she began to eat very little, stopped drinking and started a job. She entered a relationship at the age of 18. During that time, she described herself as happy, her eating habits improved and alcohol consumption markedly reduced. However, a year ago, she had a miscarriage and her relationship ended soon after. She became depressed, started experiencing panic attacks and took an overdose of antidepressants in conjunction with alcohol as an act of despair and inability to cope with current stress and as a consequence of that she began dieting again. Past psychiatric history included diagnosis of AN (F 50.0 ICD 10) a month prior to admission, bulimia and alcohol misuse. Family history included ischemic heart disease and strokes with no confirmed history of hypokalaemia even though she reiterated the hereditary nature of her hypokalaemia problems. Her father has agoraphobia and uses alcohol as a coping mechanism for anxiety. She lived on her own in a house close to her mother, was unemployed, consumed alcohol regularly, smoked 8 to 10 cigarettes/day, practised vigorous exercise, and abused laxatives and diuretics.

National Institute for Health and Care Excellence (NICE) guidelines recommend that patients with AN requiring in-patient treatment should be admitted to a setting that can provide the skilled implementation of refeeding with careful physical monitoring (particularly in the first few days of refeeding), in combination with psychosocial interventions [3]. On admission, she was monitored for refeeding syndrome, a term which encompasses multiple metabolic disturbances that occur as a result of reinstitution of nutrition to patients who are starved or severely malnourished [4]. Her risk of refeeding syndrome was assessed by the local dietician and classified as high (BMI<16kg/m^2^, very little nutritional intake for more than 10 days prior to admission, history of alcohol abuse and pre-existing low potassium) following guidelines of the National Institute for Clinical Excellence for Nutritional Support in Adults [5] and those of Management of Really Sick Patients with Anorexia Nervosa (MARSIPAN) [5,6]. The key tasks of the in-patient medical team was to safely refeed her avoiding refeeding syndrome caused by too rapid refeeding and at the same time avoiding underfeeding syndrome caused by too cautious rates of refeeding. The nutritional support was commenced at 20kcal/kg/day with a view to increasing levels slowly to meet full requirements in 5 to 7 days.

In line with MARSIPAN recommendations 2010, physical investigations included baseline full blood count, urea and electrolytes, phosphate, calcium, magnesium, albumin, C-reactive protein, liver function tests, glucose, zinc, copper, selenium, iron profile, vitamin B12 and folate, vitamin A/E, vitamin D, thyroid function, weight – early morning weight and ECG. Daily urea and electrolytes, phosphate, calcium, magnesium and ECG were performed for 1 week then twice weekly.

**Figure 1:**
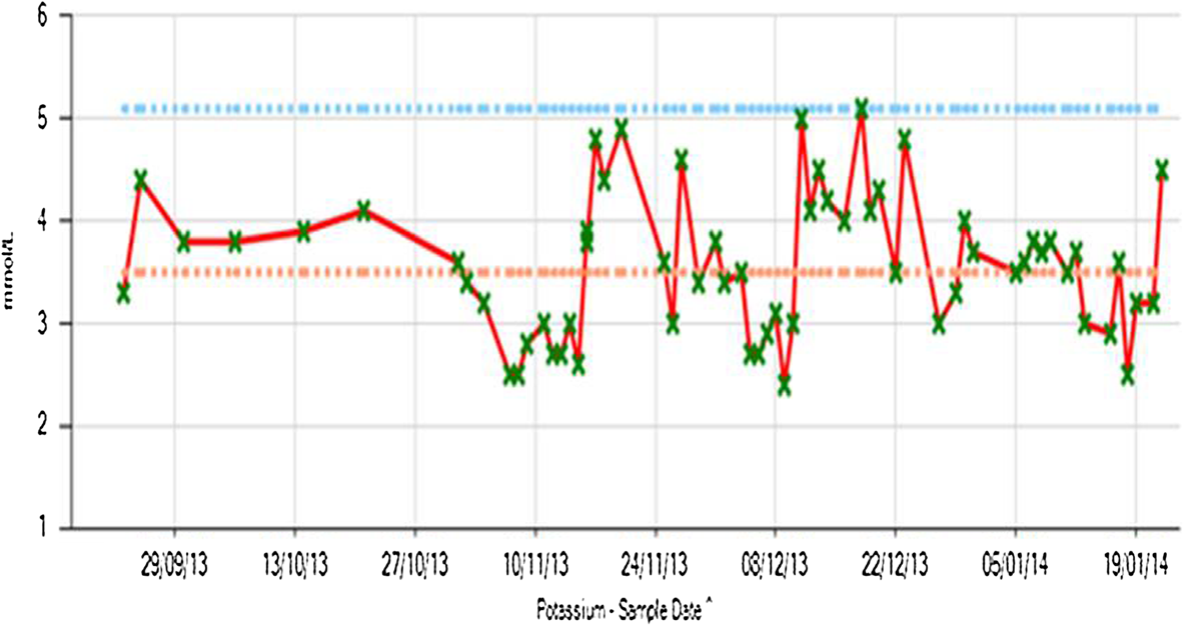
Diagram showing fluctuation in potassium levels.

After 6 months of admission, she still demonstrated bradycardia (55 beats per minute) despite improved nutritional and fluid intake. Once when she was granted leave, she admitted binge drinking and taking diuretics. Due to her distorted body image, fluctuating capacity for treatment, poor compliance with medications, laxative and diuretics abuse that led to life-threatening hypokalaemia combined with alcohol misuse, her leave was cancelled and she was detained under Section 3 of Mental Health Act 1983.Throughout her admission, she exhibited a fluctuating potassium level (2.5 to 5.0mmol/L; see Figure 1). Once on the unit, she was put on Sando-K® (potassium supplement; potassium bicarbonate and potassium chloride) but her potassium level still tended to drop. On one occasion when she became normokalaemic, supplementation was stopped and she was put on observations. After 24 hours of 1 to 1 observation by ward staff including fluid input and output monitoring as well as search of her bedroom, she was again found to be hypokalaemic in spite of no evidence to suggest laxative or diuretic abuse.

She was prescribed ferrous sulphate 200mg twice daily and an antidepressant in the form of sertraline tablet 50mg in the morning and 100mg at night. As part of her treatment for AN she was given regular supplements: thiamine, Vitamin B Compound Strong (nicotinamide, pyridoxine hydrochloride, riboflavine and thiamine mononitrate), Sanatogen® (vitamin supplement and Adcal D3® (calcium carbonate and vitamin D3).

During her time on the ward, she demonstrated overall improvement with occasional rises in her weekly BMI far above NICE recommendations as a result of abnormal fluid consumption which required implementation of fluid monitoring charts in the longer term. Gradually she became more insightful about her diagnosis and compliant with her meals and her BMI reached 18. Her management plan since admission included full meal plan, work around management of hypokalaemia, several psychotherapeutic interventions such as motivational interviewing, psychoeducation, mindfulness, body image, and relapse prevention. She appeared be hypokalaemic despite nutritional interventions on top of taking Sando-K® (potassium supplement; potassium bicarbonate and potassium chloride) tablets. She underestimated the risks to her own health and was ambivalent about being an in-patient any further. She continued to deny any purging behaviours and appeared distressed and upset by fluctuations in her potassium levels. She requested to have her hypokalaemia investigated successfully to get more clarity behind her persistent hypokalaemia.

Hypokalaemia is defined as a serum potassium level below 3.5mmol/L and is commonly classified as mild (3.1 to 3.5mmol/L), moderate (3.0 to 3.5mmol/L) and severe (<2.5mmol/L) [7]. It is a disorder with a myriad of causes and is a common finding in the general population but commoner in the hospital population with a prevalence of 5% [5]. Most causes of hypokalaemia are evident from the clinical picture and routine plasma biochemistry evaluations and there is little need to resort to further more complex and expensive biochemical tests. However, in patients for whom the cause of hypokalaemia is not obvious, determination of potassium and chloride concentrations in random urine concentrations coupled with further clinical investigations often clarify the pathogenesis of the disturbance. If serum potassium was severely low (<2.5mmol/L) or symptoms of severe hypokalaemia were present (muscle weakness, syncope or palpitations), the patient would need to be evaluated.

Multiple tests were organised to find a physical cause for our patient’s chronic hypokalaemia. On physical examination, enlargement of parotid glands was recorded. An ECG carried out indicated small T waves and prominent U waves. A renal ultrasound revealed normal sonographic appearances of both kidneys. Her 9 a.m. cortisol was normal. Fruitful areas of clinical investigations of hypokalaemia included estimation of potassium concentration in the urine. In the presence of hypokalaemia, a urinary potassium concentration at the level of 112mmol/L implies increased renal excretion of potassium. This can suggest an extrarenal defect in either potassium metabolism or distribution [8]. Her urine chloride was very low (<20mmol/L), urine pH was 8.28, and urinary bicarbonate was found to be 111mmol/L indicating high possibility of gastrointestinal losses through vomiting. These results are consistent with hypokalaemic metabolic alkalosis secondary to purging. The diuretic screen was negative. Her urinary amino acids were within normal range ruling out a renal tubular disorder. Her urine was analysed for electrolytes, amino acids, and diuretics. Venous blood gas analysis showed metabolic alkalosis with pH 7.5, bicarbonate of 35.1 and base excess of 11.

Diuretics remain the commonest cause of renal loss of potassium but rarer causes such as renal tubular acidosis often associated with autoimmune conditions such as Sjögren’s syndrome or hereditary renal tubular disorders such as Bartter’s or Gitelman’s syndrome needed to be excluded [3]. Assessment of serum magnesium was crucial because hypokalaemia and hypomagnesaemia coexist and treatment of hypokalaemia is unlikely to be successful without correction of the hypomagnesaemia. However, the patient presented with a normal magnesium level. As a patient with persistent hypokalaemia, apart from renal assessment, she needed special endocrine assessments which included renin to aldosterone ratio (to investigate for aldosteronism), thyroid function tests (to investigate for thyrotoxic periodic paralysis) but all appeared normal. A test for amylasemia was not deemed necessary by the Consultant biochemist because sufficient evidence was available to explain that her persistent hypokalaemia was due to purging.

She was assessed for symptoms of eating disorder on the Eating Disorder Examination Questionnaire (EDE-Q), obsessive compulsive symptoms on the Yale-Brown Obsessive Compulsive Scale (Y-BOCS) and depression on Hamilton Depression Scale on admission (BMI 13.5) and 3 weeks prior to discharge (BMI 18.15).

Her responses to an EDE-Q on admission indicated higher than average scores in the following test domains: shape concern with score 14 (norms 2.1), and weight concern with score 4 (norms 1.5) which went down mildly in score to 8 and 3, retrospectively after therapy. It is worth mentioning that on admission she presented with very limited insight into her eating disorder overtly disapproving AN symptoms. The Y-BOCS implemented on admission and prior to discharge appeared non-significant. However a vast reduction in overall score of both Hamilton Anxiety Scale and Hamilton Depression Scale were demonstrated, going from moderate to mild. Her score in her Hamilton Anxiety Scale decreased from 25 to 16 and her score in the Hamilton Depression Scale decreased from 27 to 8.

Neuropsychological assessment involving evaluation of both emotional and cognitive processes was conducted 4 weeks prior to discharge. No marked alterations were found in performance of social cognitive tests including perception of emotional cues on Emotion Evaluation Test, Part 1 of The Awareness of Social Inference Test (TASIT; score 26 out of 28), and emotional empathy assessed using Balanced Emotional Empathy Scale (BEES; score 51, norms 45) whereas some level of impairment in mentalising ability was elicited in her performance of the “Reading the Mind in the Eyes” test (revised version; RME; 23 out of 36). She scored low (38; norms ≤51 low alexithymia) on the Toronto Alexithymia Scale (TAS-20) and was categorised as nonalexithymic. Her performance of neurocognitive tests enabling assessment of her attentional switching and working memory (Stroop task, score 2.5 minutes/2 errors; and Trail Making Test -B, TMT-B, score 55 seconds) as well as attention per se (TMT-A, score 30 seconds) remain unremarkable.

## Conclusions

The results of the numerous investigations were explained to the patient. She continued to deny any purging behaviour. She kept reiterating to clinicians that her potassium depletion is hereditary and for that reason long-term administration of Sando-K® (potassium supplement; potassium bicarbonate and potassium chloride) tablet remained essential. Self-denial was continued until her planned discharge; however, positive behavioural change was recorded whilst her blood showed normokalaemia for a whole week prior to her discharge. At discharge, she was normokalaemic therefore her potassium supplementations were discontinued.

## Consent

Written informed consent was obtained from the patient for publication of this case report and accompanying images. A copy of the written consent is available for review by the Editor-in-Chief of this journal.

## Competing interests

All the authors declare that they have no competing interests.

## Authors’ contributions

KM was involved in conception, design, literature review and writing the first draft of the manuscript, AK was involved in acquisition of data and data analysis, KKP was the consultant in charge of the patient and was a major contributor in writing the manuscript, AN was also the consultant in charge of the patient and provided critical intellectual content. All authors read and approved the final manuscript.
